# Supplementary material for: Differences in Nanoplastic Formation Behavior Between High-Density Polyethylene and Low-Density Polyethylene
Source: Molecules. 2025 Jan 17;30(2):382. doi: 10.3390/molecules30020382 (PMC11767353; doi:10.3390/molecules30020382)
Supplement: Supplementary file 1 [file molecules-30-00382-s001.zip › molecules-3314178-supplementary.pdf]

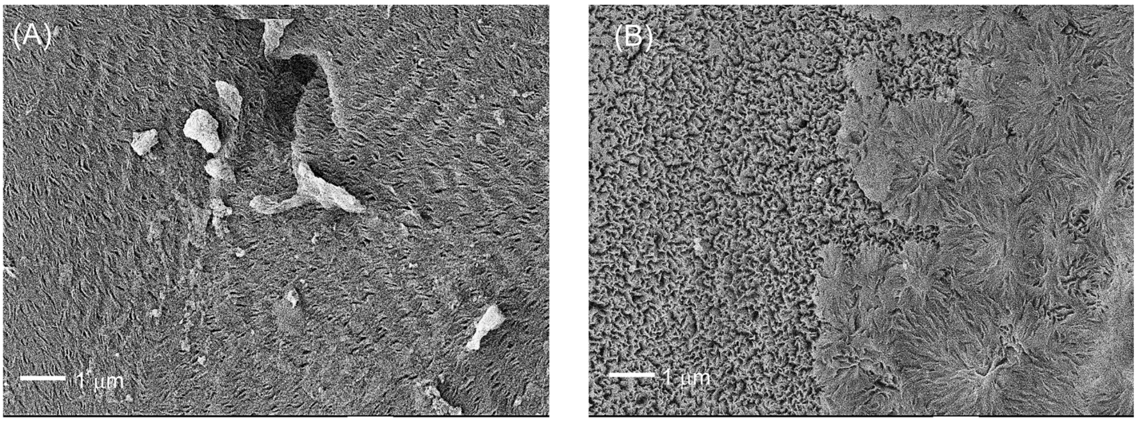

Figure S1. SEM photographs of HDPE samples degraded for 12 days: A: Before detachment (×10,000). B: After detachment (×10,000).

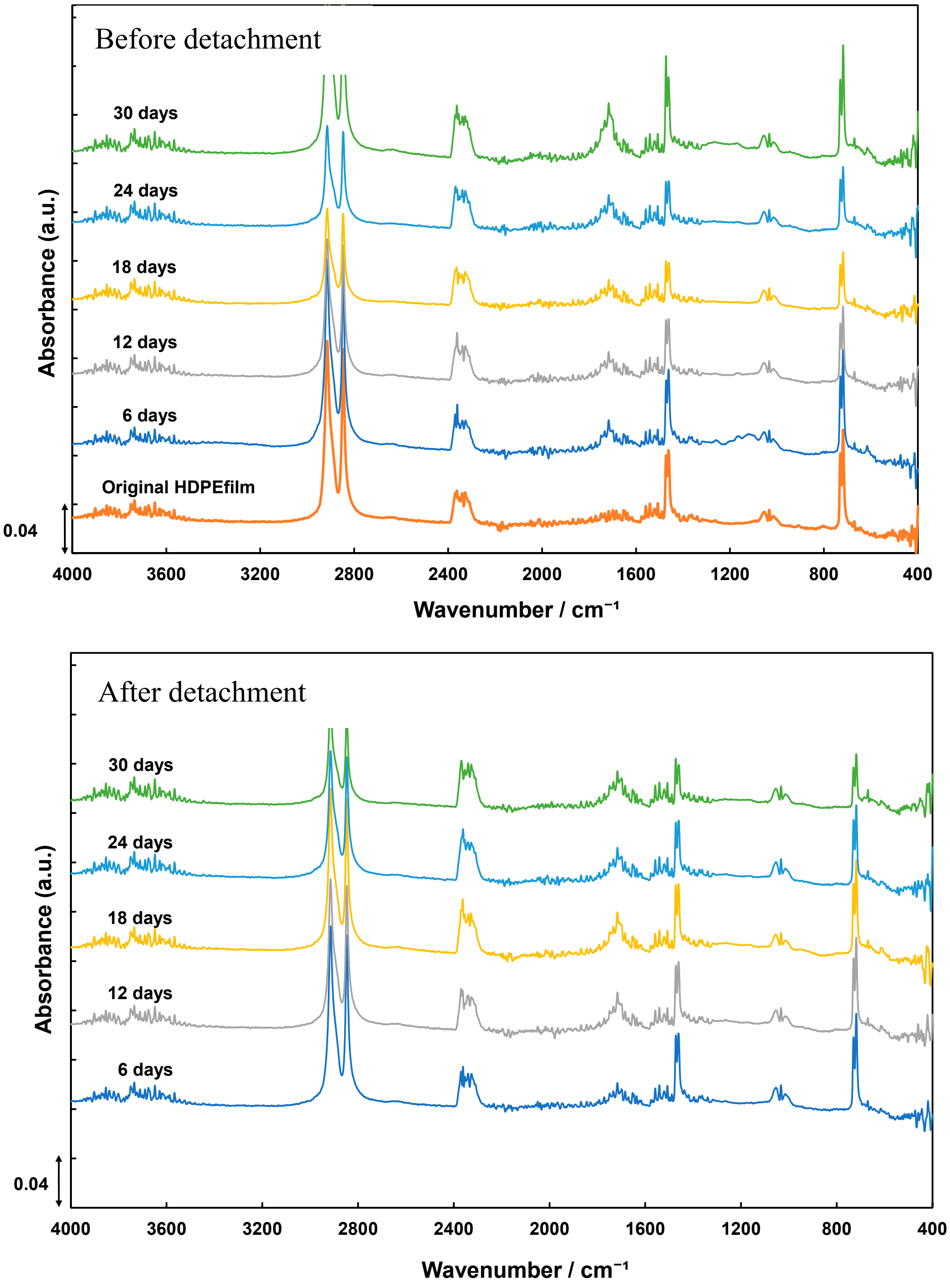

Figure S2. FT-IR spectra of degraded HDPE samples before and after detachments.

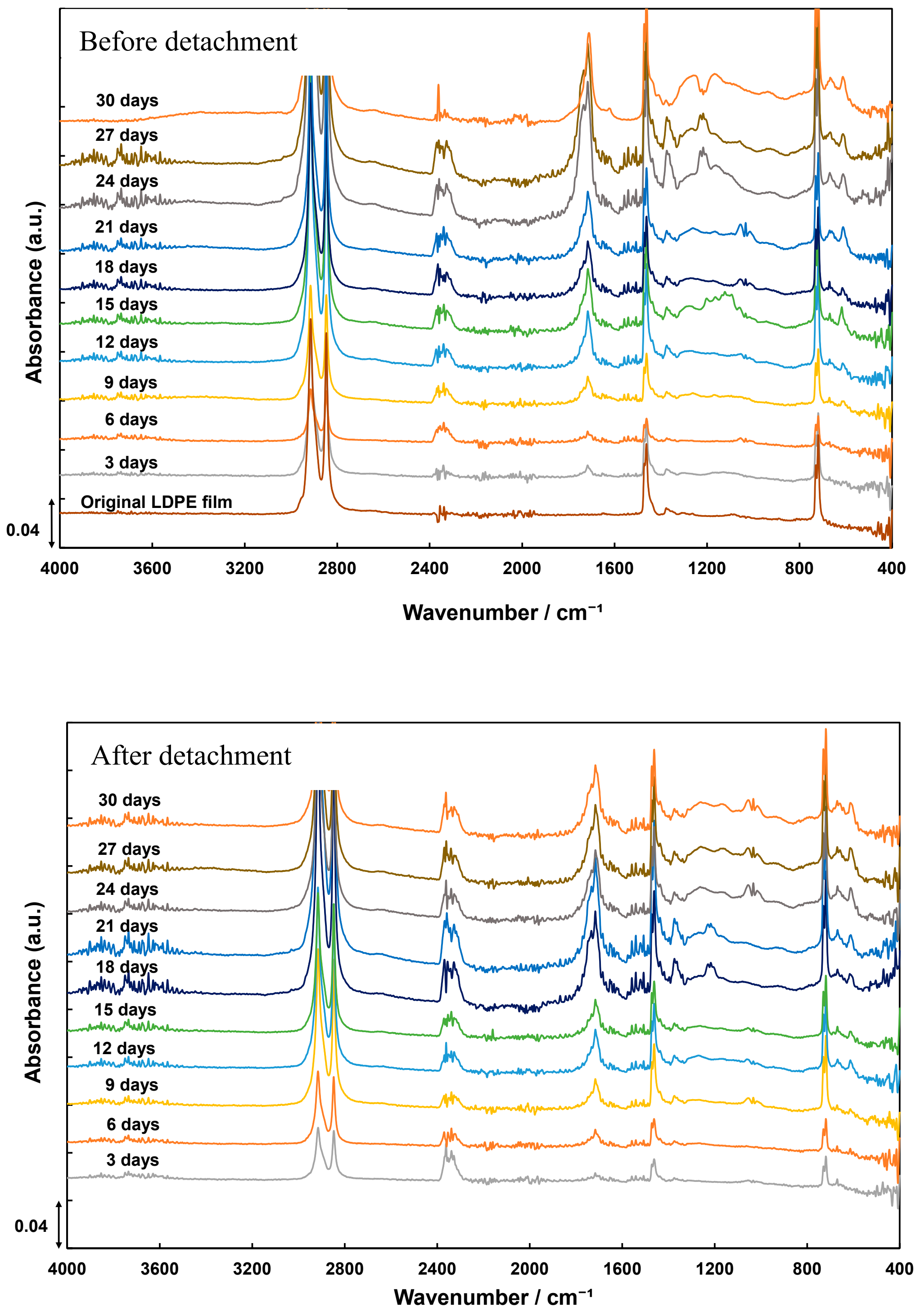

Figure S3. FT-IR spectra of degraded LDPE samples before and after detachments.

Table S1 Melting temperature (T_m), fusion enthalpy (ΔH) and crystallinity (χ_c) of before and after detachment parts of degraded HDPE samples with accelerated degradation method.

	Degradation time / days	T_m / °C	ΔH (J/g)	χ_c (%)
Before detachment	0	125	164	57
	6	124	157	54
	12	125	160	55
	18	123	158	54
	24	122	120	41
	30	121	156	54
After detachment	6	126	148	51
	12	123	151	52
	18	123	147	51
	24	124	158	54
	30	119	157	54

Table S2 Melting temperature (T_m), fusion enthalpy (ΔH) and crystallinity (χ_c) of before and after detachment parts of degraded LDPE samples with accelerated degradation method.

	Degradation time / days	T_m / °C	ΔH (J/g)	χ_c (%)
Before detachment	0	110	137	47
	3	104	110	38
	6	103	101	35
	9	102	110	38
	12	102	106	36
	15	102	113	39
	18	102	113	39
	21	101	113	39
	24	101	108	38
	27	101	106	36
	30	101	112	37
After detachment	3	104	103	36
	6	103	116	40
	9	102	107	37
	12	102	103	35
	15	101	107	37
	18	103	113	39
	21	104	113	39
	24	101	110	38
	27	101	105	36
	30	101	106	37
